# Advanced Taste Sensors Based on Artificial Lipids with Global Selectivity to Basic Taste Qualities and High Correlation to Sensory Scores

**DOI:** 10.3390/s100403411

**Published:** 2010-04-08

**Authors:** Yoshikazu Kobayashi, Masaaki Habara, Hidekazu Ikezazki, Ronggang Chen, Yoshinobu Naito, Kiyoshi Toko

**Affiliations:** 1 Intelligent Sensor Technology, Inc., 5-1-1 Onna, Atsugi-shi, Kanagawa 243-0032, Japan; E-Mail: taste.sensor@insent.co.jp; 2 Information Science and Electrical Engineering, Kyushu University, Fukuoka 819-0395, Japan; E-Mail: toko@ed.kyushu-u.ac.jp; 3 System Life Sciences, Kyushu University, Fukuoka 819-0395, Japan; E-Mail: habara@belab.ed.kyushu-u.ac.jp

**Keywords:** taste sensor, artificial lipid, CPA value, global selectivity, high correlation to human sensory score

## Abstract

Effective R&D and strict quality control of a broad range of foods, beverages, and pharmaceutical products require objective taste evaluation. Advanced taste sensors using artificial-lipid membranes have been developed based on concepts of global selectivity and high correlation with human sensory score. These sensors respond similarly to similar basic tastes, which they quantify with high correlations to sensory score. Using these unique properties, these sensors can quantify the basic tastes of saltiness, sourness, bitterness, umami, astringency and richness without multivariate analysis or artificial neural networks. This review describes all aspects of these taste sensors based on artificial lipid, ranging from the response principle and optimal design methods to applications in the food, beverage, and pharmaceutical markets.

## 1. Introduction

Taste evaluation is gathering attention worldwide in many fields, such as foods, beverages, and pharmaceuticals. Sensory evaluation and chemical analysis are commonly used to evaluate taste qualities of the products. However, sensory evaluation using a panel of tasters is susceptible to human physical and psychological conditions as well as individual preference, making panel scores highly subjective. In contrast, chemical analysis by high-performance liquid chromatography (HPLC) offers quantitative data that cannot be explained in terms of overall taste because the data cover each taste substance in the food. Finally, chemical analysis cannot detect taste-substance interactions, such as synergistic and suppression effects.

Clearly taste evaluation needs a new quantitative and objective method. The so-called “electronic tongue” is one solution researched since the mid-1990s [1–7]. It uses either ion-specific electrodes [1–5], or pulse voltammetry techniques [6,7], which provide information on the sample composition using multivariate analyses or artificial neural networks. Although such techniques can offer effective quality control, they are inappropriate for development of foods, beverages, and pharmaceuticals because classification based on ion species does not evaluate actual taste. We still need an objective method for evaluating the taste of samples.

Following our basic research on lipid/polymer membranes [8–22], we developed a Taste Sensing System correlated with the taste perception of living organisms by using artificial lipids as a transducer for multichannel taste sensors [23–31]. Further improvements led to successful development of advanced taste sensors capable of evaluating saltiness, sourness, bitterness, sweetness, umami and astringency. These taste sensors are based on very different concepts from the electronic tongue and feature global selectivity and high correlation with human sensory score. They offer satisfactory taste results closer to human sensory evaluation while eliminating the need for multivariate analyses and artificial neural networks. This review describes all aspects of these taste sensors based on artificial lipid, ranging from the response principle and optimal design methods to applications in the food, beverage, and pharmaceutical markets.

## 2. Taste Sensors

### 2.1. Using artificial lipid-based membrane

There are many taste substances but the sense of taste has five qualities: saltiness, sourness, bitterness, sweetness, and umami (savoriness) [32,33]. These qualities are called basic tastes and each plays an important role for humans. Saltiness, which is caused mainly by ionic materials, is a good indicator of electrolyte balance in foods; sourness, which is produced by organic acids, signals decomposition; bitterness, which is often considered distasteful, prevents intake of poisonous materials; umami, which is evoked by some amino acids, provides information on the presence of amino acids; sweetness, which is produced by sugars or sugar alcohols, has a role in indicating nutrient sources. Astringency, which is produced mainly by tannins, is sometimes considered a taste quality in the broad sense [34–36].

**Figure 1.**
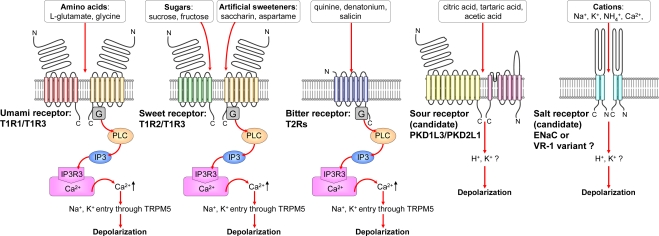
Taste receptors for five basic taste qualities and signal transduction pathways. G, GTP-binding protein; PLC, phospholipase C-type β 2; IP3, inositol 1,4,5-trisphosphate; IP3R3, 1,4,5-trisphosphate receptor type 3; TRPM5, transient receptor potential cation channel, subfamily M, member 5. For details, see references [38–49].

The “fluid mosaic model” was proposed to explain the structure of biological membranes [37] in the early 1970s. In this model, proteins move in a sea of lipid molecules on cell membranes, including taste cells. Recent advancements have identified the taste receptor cells on the human tongue for the five basic tastes [38–44]; their signal pathways are shown in Figure 1. There are about 100 taste receptor cells composed of a lipid bilayer in the taste buds of the human tongue. They are distributed across three types of papillae: circumvallate, foliate, and fungiform, located at the back, posterior lateral edge, and anterior of the tongue. Umami, sweet and bitter compounds are received by seven transmembrane domain receptors interacting with intercellular G proteins, or G protein-coupled receptors (GPCRs). Several types of GPCRs (T1R1, T1R2, T1R3, and T2Rs) are involved in taste transduction. The T1R1+T1R3 heteromer, T1R2+T1R3 heteromer, and T2Rs GPCRs function as umami, sweet, and bitter receptors, respectively [38–40]. In contrast, stimuli evoked by sour materials are thought to be perceived via a candidate sour receptor called the PKD1L3-PKD2L1 channel, which is a transient receptor potential (TRP) family member [41,42]. The salt receptor epithelial sodium channel (ENaC), which is an amiloride-sensitive Na^+^ channel, allows Na^+^ ions to enter the taste-cell membrane. In addition, the amiloride-insensitive channel vanilloid receptor-1 variant, functions as a non-selective cation channel [43,44]. However, it is still not known whether these channels serve as a salt receptor. All tastes are detected and perceived via these taste receptors, which mediate signal cascades through second messenger molecules [45–49].

Current research shows that the taste-receptor proteins play a key role in sensing taste but it may be difficult to create artificial protein-based taste sensors because proteins have low chemical and thermodynamic stability. However, research in the mid-1970s [50,51] showed that the membrane potential of filter paper impregnated with all the lipids extracted from bovine tongue epithelium changed like that of a living taste receptor cell in response to salts and acids. What is important is how the five basic taste qualities are discriminated and their intensities are quantified. As described later sensors using lipid membranes provide satisfactory results. Following this early lead, we started developing taste sensors using artificial lipids. Most lipid molecules are composed of hydrophobic and hydrophilic groups, so lipids are thought to interact with various taste materials via electrostatic and hydrophobic interactions. After more than 10 years in R&D [8–28], the first commercial *SA401* Taste Sensing System was introduced in Japan in 1993. However, taste sensors at that time had inadequate selectivity for evaluating taste objectively.

We launched new research in 1999 to make a breakthrough in taste sensors by achieving higher selectivity for each taste [52–54], especially bitterness and astringency, which are difficult to evaluate by conventional chemical analysis. We found that sensor selectivity for each taste is improved by modulating both the hydrophobic interaction between the taste sensor and bitter or astringent substance [52,53] and the membrane charge density [54] (See Sections 3.1 and 3.2 for more details). Breakthrough innovation from the perspective of sensor engineering rather than biology suggests four requirements are needed to achieve objective taste evaluation: (1) The taste sensor must respond consistently to the same taste like the human tongue (global selectivity); (2) The taste sensor threshold must be the same as human taste threshold; (3) There must be a clearly defined unit of information from the taste sensor; and (4) The taste sensor must detect interactions between taste substances (see Section 3). Our current taste sensors satisfy all the requirements. High correlation with human sensory score means taste sensors respond to samples even at different intensity just like the human gustatory sense. With these unique features, advanced taste sensors can evaluate taste objectively.

### 2.2. Reagents

**Figure 2.**
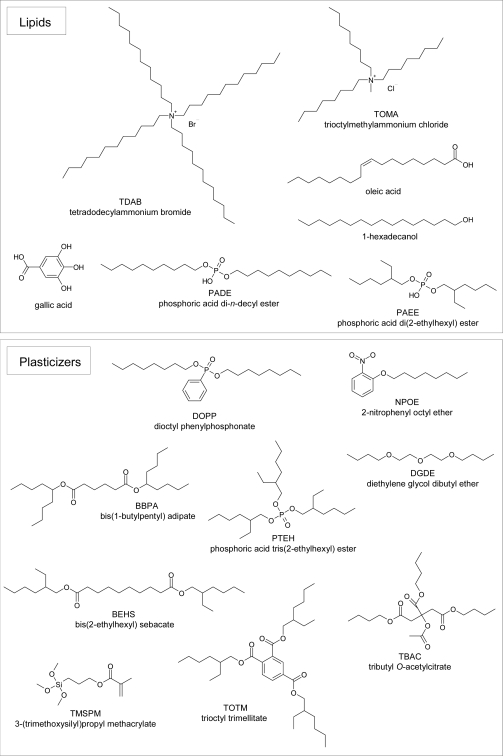
Chemical structures of artificial lipids and plasticizers (Reprinted with permission from the Institute of Electrical Engineers of Japan [55]).

The artificial-lipid sensors were made using tetradodecylammonium bromide (TDAB), trioctylmethylammonium chloride (TOMA), oleic acid, 1-hexadecanol, gallic acid, phosphoric acid di-*n*-decyl ester (PADE), and phosphoric acid di(2-ethylhexyl) ester (PAEE). Dioctyl phenyl-phosphonate (DOPP), 2-nitrophenyl octyl ether (NPOE), bis(1-butylpentyl) adipate (BBPA), bis(2-ethylhexyl) sebacate (BEHS), phosphoric acid tris(2-ethylhexyl) ester (PTEH), tributyl *O*-acetylcitrate (TBAC), 3-(trimethoxysilyl)propyl methacrylate (TMSPM), diethylene glycol dibutyl ether (DGDE), and trioctyl trimellitate (TOTM) were used as the plasticizer. The polymer support was polyvinyl chloride (PVC). Tetrahydrofuran (THF) was used as the preparation solvent. The TDAB, NPOE, BBPA, BEHS, DGDE, TMSPM, TOTM, and THF were purchased from Sigma-Aldrich, USA. The TOMA, PTEH, TBAC, PADE, PAEE, oleic acid, and gallic acid were purchased from Tokyo Chemical Industry Co., Ltd., Tokyo, Japan. The 1-hexadecanol and PVC were purchased from Wako Pure Chemical Industries, Ltd., Osaka, Japan. The DOPP was purchased from Dojindo Laboratories, Kumamoto, Japan. The chemical structures of the lipid and plasticizers are shown in Figure 2.

### 2.3. Fabrication

Various amounts of lipid and plasticizer were mixed for 1 hour in 10 mL of THF, depending on the taste sensor type. The mixture was dried in a Petri dish at room temperature for 3 days to form the transparent membrane. The membrane was attached to the sensor surface using a solution of 800 mg of PVC and 10 mL of THF.

### 2.4. Measurement system

**Figure 3.**
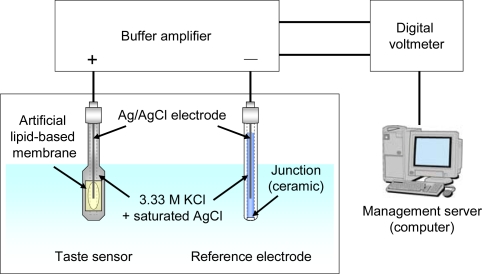
Diagram of taste sensing system (Reprinted with permission from the Institute of Electrical Engineers of Japan [55]).

Figure 3 shows a diagram of the taste sensing system with the taste sensor acting as the working electrode. The Ag/AgCl electrode with a single ceramic junction is the reference electrode. A solution containing 3.33 M KCl and saturated AgCl was used as the inner solution for the sensors and reference electrode. These electrodes were conditioned for 2 days in a solution of 30 mM KCl and 0.3 mM tartaric acid before measurement.

**Figure 4.**
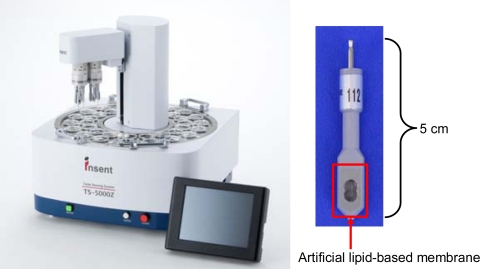
*TS-5000Z* Taste Sensing System. Left: TS-5000Z, Right: Taste sensor.

Taste Sensing System models *SA401*, *SA402*, and *SA402B* were sold in Japan in 1993, 1996, and 2000, respectively. Figure 4 is a photograph of the fourth *TS-5000Z* model composed of a sensor unit and management server. Up to 8 sensors can be connected to the unit, providing data on taste qualities, such as sourness, saltiness, umami, bitterness, astringency, and richness.

### 2.5. Mechanism of taste sensor response

Based on classical Gouy–Chapman theory [56,57], it is well known that an electrical double layer is formed on a charged membrane. To clarify the electrical characteristics of the lipid/polymer membrane in response to taste substances, first, we calculated the theoretical charge density at the membrane surface using Gouy–Chapman theory and Poisson–Boltzmann equation [58,59]. Then, we investigated the lipid/polymer membrane’s responses to sodium chloride (salty), hydrochloric acid (sour), monosodium glutamate (umami), and quinine hydrochloride (bitter), and compared the experimental and calculated theoretical results [60,61].

**Figure 5.**
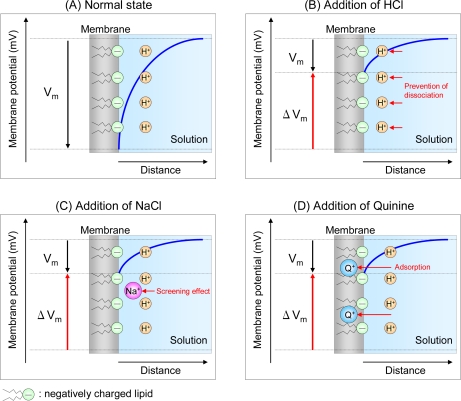
Diagram of response mechanisms of negatively charged membrane to sour, salt, and bitter taste substances. V_m_, membrane potential; ΔV_m_, change in membrane potential (sensor output); H^+^, proton dissociated from lipid molecule; Na^+^, sodium ion; Q^+^, quinine ion. The blue curve represents the change in electrical double layer with distance.

The mechanism of taste sensor response can be explained by our findings. Figure 5 shows the response mechanisms of a negatively charged lipid/polymer membrane to three taste substances.

When the artificial lipid-based membrane is immersed in an aqueous solution, an electrical double layer is formed at the membrane surface by dissociation of acid groups of lipid molecules, causing membrane potential (Figure 5A). The response to sour materials shows that the response of a negatively charged membrane to HCl is in good agreement with the theoretical result. Therefore, sour substances prevent lipid molecule dissociation, changing the membrane potential [60] (Figure 5B). The sensor response to NaCl is also in good agreement with the theoretical result, demonstrating that salt substances affect the electrical double layer at the sensor surface (Figure 5C), causing a change in the membrane potential (called screening effect) [60,61]. The sensor response to quinine hydrochloride is smaller than the theoretical result, suggesting a different sensor response mechanism than to NaCl and HCl [60]. Consequently, we investigated the amount of quinine hydrochloride in a negatively charged membrane immersed in 1 mM quinine hydrochloride for 1 hour using electron spectroscopy for chemical analysis (ESCA) [62]. There is an N1s peak at 400 eV, indicating nitrogen in the membrane. Since there is no nitrogen in any membrane component, this result implies adsorption of quinine hydrochloride into the membrane. These results suggest bitter materials are adsorbed on the hydrophobic part of the membrane and cause a change in membrane potential by changing the charge density (Figure 5D) [60–62]. The sensor response to monosodium glutamate (MSG) is inconsistent with the theoretical result, again indicating a different response mechanism than to NaCl and HCl [60]. Although ESCA analysis shows no N1s peak [62], the fact that the negative charge of the sensor increases with MSG concentration suggests some interaction with MSG [62,63]. Therefore, we believe MSG has such an extremely slight hydrophobic interaction with the lipid membrane that MSG is easily desorbed from the membrane by the rinsing with pure water before ESCA analysis. As one explanation, MSG is thought to interact with the negative lipid using the positively charged amino group, while the negatively charged carboxyl group makes the membrane potential more negative [62].

### 2.6. Measurement procedure

**Figure 6.**
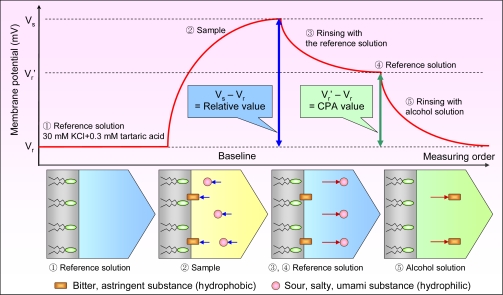
Measurement procedure. *Note: The reference solution should be tasteless compared to the measured sample. Therefore, the reference solution should have a lower concentration for samples with extremely low concentrations (e.g., 1 mM KCl in Figure 10).

Figure 6 shows the measurement procedure with the change in membrane potential over time. First, the sensor is immersed in the reference solution of 30 mM KCl and 0.3 mM tartaric acid to obtain the membrane potential, V_r_. The reference solution in this system has almost no taste and mimics human saliva. Second, the sensor is immersed in the sample solution to obtain the potential, V_s_. Third, the sensor is rinsed lightly with the reference solution. After rinsing, it is immersed in the reference solution again to obtain the potential, V_r_’. As shown in Figure 6, the difference in potential (V_s_ – V_r_), called the relative value, should approximate the initial taste at sensory evaluation, including sourness, saltiness, and umami. The difference in potential (V_r_’ – V_r_) called CPA (Change of membrane Potential caused by Adsorption) provides data on the adsorption of bitter and astringent substances by the artificial lipid-based membrane [52,54]. This value is significant for evaluating bitterness and astringency, because the corresponding taste substances are thought to be adsorbed strongly on the human tongue. Finally, the sensor is rinsed well in alcohol solution to remove adsorbed substances before measuring the next sample.

## 3. Taste Sensor Design

As mentioned, there are four requirements for objective taste evaluation: (1) The taste sensor must respond consistently to the same taste like the human tongue (global selectivity); (2) The taste sensor threshold must be the same as the human taste threshold; (3) There must be a clearly defined unit of information from the taste sensor; and (4) The taste sensor must detect interactions between taste substances (see Subsection 5.3). Item (1) eliminates use of multivariate analyses, making it easy to interpret sensor output data with regard to taste quality. Item (2) provides results mimicking the human gustatory sense. Item (3) is essential for objective evaluation of taste. For example, data cannot be interpreted as taste quality or intensity if it is unclear what the graph axes explicitly represent in principal component analysis (PCA). Therefore, if the origins of all the samples are unknown, it is impossible to interpret both taste quality and intensity in the analysis. Item (4) enables sensor data to be consistent with sensory evaluation scores even when interactions between taste materials increase or decrease taste intensity. When the first Taste Sensing System was launched in 1993, all taste sensors had low taste selectivity, causing difficulties in evaluating samples with unknown taste. Although the first Taste Sensing System used PCA to classify samples based on information from the low-selectivity sensors, the result was just the sum of less taste information.

We found that physicochemical properties vary with the types of taste substances. Table 1 shows the physicochemical properties of four taste qualities [54]. Salts like NaCl are easily hydrated in an aqueous solution, so they are hardly adsorbed by the hydrophobic part of lipid molecules; the threshold of taste for these materials is relatively high because they are essential to life. Sour substances like acetic acid also have no ability for adsorption by the hydrophobic part of a lipid molecule because they are also easily hydrated in the solution, while their taste threshold is quite low because sourness is a signal to indicating food decomposition. Bitter materials are slightly soluble in the solution due to their high hydrophobicity, and their taste threshold is very low, because bitterness is generally produced by toxic substances, which has high survival advantages for easy recognition at the lowest concentrations. Umami substances like MSG or peptide have a slight aftertaste, sometimes called “richness”. This may be due to their slight hydrophobicity, helping adsorption on the tongue and causing a lasting slight aftertaste. Therefore, hydrophobicity strength should be recognized “low” among taste qualities.

Although this classification makes little sense from the biological viewpoint, it has great significance in sensor technology. By focusing on these properties, we propose two methods to improve the selectivity and sensitivity of taste sensors by modulating the electric charge density of the membrane and the hydrophobicity of the membrane surface.

### 3.1. Optimizing electric charge density of membrane

**Figure 7.**
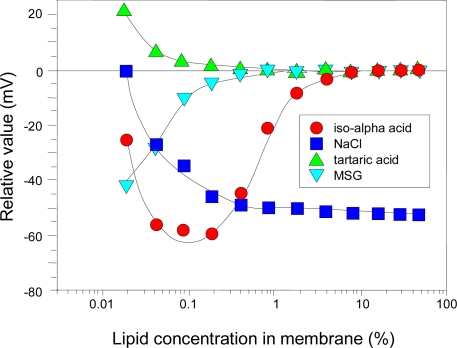
Relationship between lipid concentration in membrane and relative values of bitterness sensor. The concentrations of each sample are: iso-alpha acid, 0.01 vol%; NaCl, 300 mM; tartaric acid, 2.7 mM; MSG, 10 mM. All samples include 30 mM KCl and 0.3 mM tartaric acid as supporting electrolyte (Reprinted with permission from the Institute of Electrical Engineers of Japan [53]).

To meet the first requirement for global selectivity (i.e. like the human tongue, the taste sensor must respond consistently to the same taste), modulating electric charge density of the membrane is quite effective for improving selectivity and sensitivity to bitter and astringent materials [53]. Figure 7 shows the relationship between lipid concentration in membrane and relative value for a bitterness sensor composed of the positively charged lipid, tetradodecyl ammonium bromide (TDAB), and the plasticizer, 2-nitrophenyl octyl ether (NPOE). The sensor is very sensitive to bitter materials, such as iso-alpha acid, which is negatively charged in a solution. As shown in Figure 7, the relative value for NaCl increases negatively with the lipid concentration due to the screening effect of the electrolyte Cl^−^ anions.

Relative values for both tartaric acid (acidic) and MSG (alkaline) shift to zero as the TDAB concentration increases because TDAB functions as an anion exchanger, so the sensor does not respond to H^+^ cations generated from sour or umami substances. Intriguingly, the sensor relative value shows a non-linear response for iso-alpha acid perhaps because, unlike other taste substances, iso-alpha acid causes a change in electric potential by adsorption onto the membrane surface, which then causes the non-linear relative value.

**Figure 8.**
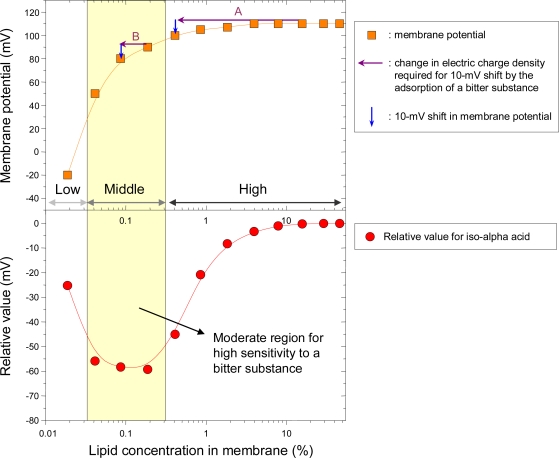
Relationship between lipid concentration in the membrane and membrane potential and the relationship between lipid concentration in the membrane and relative value for iso-alpha acid (Reprinted with permission from the Institute of Electrical Engineers of Japan [53]).

To better understand why the relative value for the bitter substance exhibits a non-linear response, Figure 8 shows the relationship between lipid concentration in the membrane and membrane potential (top figure), as well as the relationship between lipid concentration in the membrane and relative value for iso-alpha acid (bottom figure) taken from Figure 7. In the top figure, the membrane potential is the reference solution potential, V_r_ (Figure 6). The potential increases rapidly at low TDAB concentrations, but plateaus at higher TDAB concentrations. As mentioned in Subsection 2.5, adsorption of bitter substances on the hydrophobic part of sensor membrane changes the charge density, causing the relative value.

So how much change in charge density is needed to cause a shift in the membrane potential? First, in the high-concentration region in Figure 8, inducing a 10-mV shift in the membrane potential requires a dramatic change in the charge density indicated by purple arrow A. However, such a dramatic change is impossible because only a very slight amount of bitter substance is adsorbed. Therefore, little or no relative value is obtained, as shown in the same region in the bottom figure in Figure 8. Second, in the middle-concentration region, a slight change in the charge density, which is indicated by purple arrow B, can easily induce a 10-mV shift in the membrane potential, producing high sensitivity to a bitter substance, as shown in the same region in the bottom figure. This region can be considered moderate for high sensitivity to a bitter substance. Last, in the low-concentration region, there is no lipid to adsorb a bitter substance, leading to low sensitivity, as shown in the same region in the bottom figure.

These findings suggest that achieving high sensitivity to bitter or astringent substances requires incorporating appropriate amounts of lipid in the membrane to cause the maximum shift in membrane potential by changing the electric charge density.

Salty substances change the electric potential of the membrane due to the screening effect of ions from the substances; sour substances affect electric potential by dissociating of acid groups of lipid molecules in the membrane. Therefore, sensitivity and selectivity to salty and sour substances can be achieved by incorporating more lipids in the membrane, helping reduce sensitivity to bitterness and astringency [54] as described above.

Umami substances change electric potential of the membrane due to the screening effect of ions and slight adsorption (see Subsection 2.5). Consequently, a medium amount of membrane lipid shows highest selectivity for umami [54].

### 3.2. Optimizing hydrophobicity of membrane

Another approach to meeting the first sensor requirement for global selectivity is optimizing the hydrophobicity of the membrane surface. An example of developing another bitterness sensor using this approach is described below.

**Figure 9.**
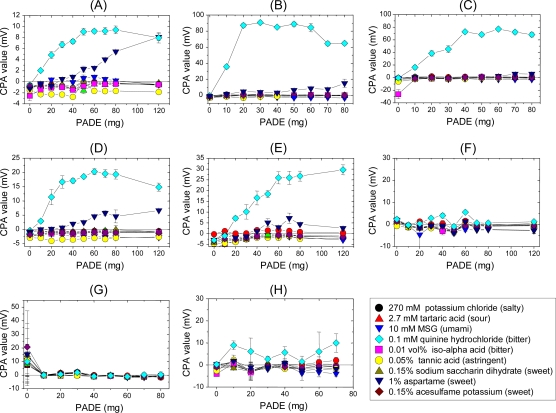
Sensor responses to basic taste substances. The x-axis represents PADE contents in the membrane, while the y-axis shows the CPA value. Data are expressed as mean ±SD (n = 4). All samples include 30 mM KCl and 0.3 mM tartaric acid as supporting electrolyte. Lipid: PADE (phosphoric acid di-*n*-decyl ester); Plasticizer: A, DOPP; B, BBPA; C, BEHS; D, PTEH; E, TBAC; F, TMSPM; G, DGDE; H, TOTM (Reprinted with permission from the Institute of Electrical Engineers of Japan [55]).

Bitter substances are sensed by the T2Rs bitter taste receptor [39,40], but are also thought to be adsorbed on the surface membrane of taste cells [64]. To control adsorption, we focused on LogD, which is known to be correlated with hydrophobicity [65–67]. Therefore, taste sensors based on 8 plasticizers with different hydrophobicity were examined for sensitivity and selectivity to several taste substances (Figure 9) [55]. The sensors with BBPA, BEHS, PTEH and TBAC plasticizers are very selective for quinine hydrochloride although all are based on PADE lipid, suggesting that hydrophobicity of the membrane significantly affects sensitivity and selectivity to bitterness produced by positively charged bitter substances. Interestingly, sensors with no lipid do not respond to bitter substances at all, even when the membrane contains any of the four plasticizers. This indicates that both substantial lipid content and a plasticizer with appropriate hydrophobicity are needed for high selectivity and sensitivity and selectivity.

## 4. Sensor Characteristics

### 4.1. Threshold of taste for basic tastes

After satisfying the first requirement for global selectivity, taste sensors can be fabricated to respond similarly to similar tastes. Table 2 lists the components of eight taste sensors, including a prototype sweetness sensor for further improvement. As shown in Table 2, there are three types of bitterness sensors: C00 for acidic bitter materials, such as iso-alpha acid found in beer [68,69]; BT0 for hydrochloride salts, including quinine hydrochloride and azelastine hydrochloride mainly used as drugs [44]; and AN0 for basic materials, such as famotidine [70]. Sweet substances, including glucose and sucrose, have no charge, so sweetness sensors based on potentiometric measurement cannot respond to sweet materials. However, recent research shows that a taste sensor incorporating the artificial lipid TDAB and plasticizer DOPP has a sensitivity of around −60 mV to sweet substances, including sucrose, glucose and fructose at 1 M concentration after immersing the sensor in a solution of 0.05% gallic acid (3,4,5-trihydroxybenzoic acid) [71–73]. Interestingly, a non-immersed sensor has no sensitivity, demonstrating that a membrane surface modified by adsorption of gallic acid interacts selectively with sweet materials. There are some remaining problems to solve: (1) The sweetness sensor GL0 also responds to salty and umami samples; and (2) The sensor GL0 has low durability partly because the adsorbed gallic acid may dissolve during the measurement. We are currently developing a sweetness sensor with higher selectivity and durability by incorporating an alternative to gallic acid in the membrane.

**Figure 10.**
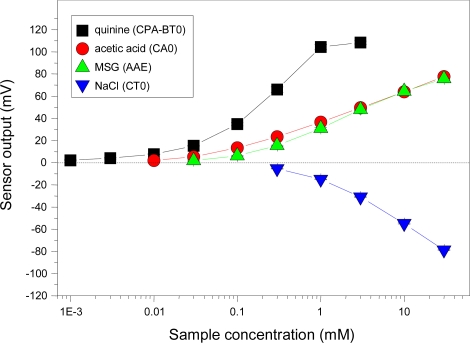
Concentration dependence of four taste sensors on taste substance. The relative values of sensors CA0, AAE, and CT0 were used for acetic acid, monosodium glutamate (MSG), and NaCl, respectively, while the CPA value of sensor BT0 was used for quinine hydrochloride. Data are expressed as mean ±SD (n = 4). All samples include 1 mM KCl as supporting electrolyte.

Taste thresholds, which differ with taste quality, increase in the order of saltiness > umami > sour > bitterness [74,75]. We investigated the concentration dependence of sensors for four basic tastes (Figure 10). The result indicates that thresholds of the sensors agree well with the human gustatory sensation, fulfilling the second requirement for a taste sensor with the same threshold as the human taste threshold. This property enables us to measure the intensity of a given taste substance. Without this property, multivariate analysis would be needed to interpret data in terms of taste.

### 4.2. Global selectivity

**Figure 11.**
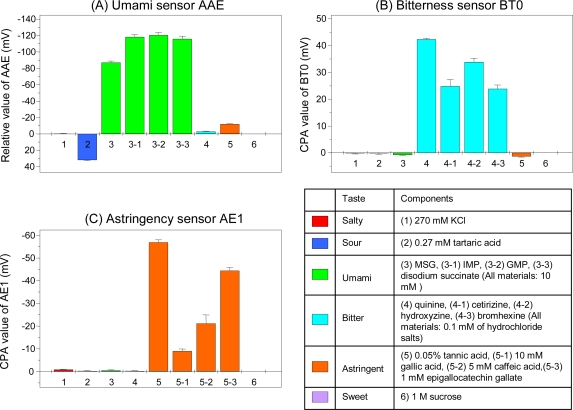
Responses of taste sensors to six tastes. Figure 11A uses the relative value of the umami sensor AAE; Figure 11B, the CPA value of the bitterness sensor BT0; Figure 11C, the CPA value of the astringency sensor AE1. Data are expressed as mean ±SD (n = 4). All samples include 30 mM KCl and 0.3 mM tartaric acid as supporting electrolyte. MSG, monosodium glutamate; IMP, disodium 5′-inosine monophosphate; GMP, disodium 5′-guanosine monophosphate.

Our taste sensors exhibit global selectivity, meeting the first requirement for a taste sensor. Figure 11 shows the responses of the bitterness sensor BT0, astringency sensor AE1, and umami sensor AAE to the basic taste qualities.

In Figure 11A, MSG (monosodium glutamate), IMP (disodium 5′-inosine monophosphate), GMP (disodium 5′-guanosine monophosphate), and disodium succinate are umami materials found in seaweeds, meats, mushrooms, and shellfish, respectively. The umami sensor AAE has a high and selective response to all these umami substances, indicating it has global selectivity to the umami taste.

In Figure 11B, the bitterness sensor BT0 responds selectively to the bitter pharmaceutical drugs quinine hydrochloride, cetirizine hydrochloride, hydroxyzine hydrochloride, and bromhexine hydrochloride, but not to any other tastes, indicating this sensor has global selectivity to bitterness. In Figure 11C, the astringency sensor AE1 responds selectively to the astringent substances tannic acid, gallic acid, caffeic acid, and epigallocatechin gallate (EGCg), indicating this sensor has global selectivity to astringency. Using these sensors, we can evaluate several taste qualities, without complex and time-consuming multivariate analysis or artificial neural networks.

### 4.3. High correlation with human sensory score

**Figure 12.**
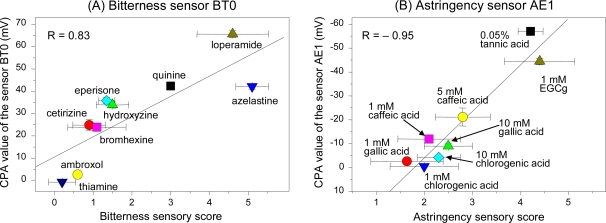
Relationship between results of taste sensors and human taste scores for similar tastes. Tastes for Figs. 12A and 12B were scored by three and eight panelists, respectively. In Figure 12A, quinine hydrochloride concentrations of 0.01, 0.03, 0.1, 0.3, and 1.0 mM were used as standards and were assigned scores of 1, 2, 3, 4, and 5, respectively. In Figure 12B, tannic acid concentrations of 0.005%, 0.011%, 0.024%, and 0.05% were used as standards and were assigned scores of 1, 2, 3, 4, and 5, respectively. The standard deviations on the x- and y-axes are the difference between the panelists’ scores and measurement error (n = 4), respectively. All samples include 30 mM KCl and 0.3 mM tartaric acid as supporting electrolyte. EGCg: epigallocatechin gallate.

Taste sensors meeting the second requirement (same threshold as human) give results closer to human sensory scores for samples with similar tastes but different taste intensities. Figure 12 shows the relationship between human taste scores and sensor evaluations for similar taste qualities.

In Figure 12A, the concentrations of all the bitter drug samples were the same (0.1 mM). However, the bitterness sensor BT0 showed different sensitivity to each sample with a high correlation (0.83) to the taste scores, suggesting that this sensor responds selectively according to bitterness intensity and does not detect just quantitative information.

In Figure 12B, the astringent samples were at different concentrations ranging from 1 to 10 mM, including 0.05% for tannic acid. The astringency sensor AE1 showed a high negative correlation (−0.95) with taste scores, indicating that it can evaluate astringency taste objectively. (This sensor responds negatively to astringent materials, so the correlation coefficient is a negative number).

These results demonstrate that artificial lipid-based membranes can function as taste sensors for objective evaluation of taste.

### 4.4. Conversion to taste information

The Weber–Fechner law states that (i) the relationship between the initial intensity for human stimuli, such as olfactory or gustatory sense, and the discrimination threshold is a constant (Weber fraction), and (ii) the relationship between the stimulus and corresponding perceived intensity is logarithmic [76,77]. The smallest detectable increment for the gustatory sense is about 20% [78]. Based on this law, sensor outputs can be converted to “taste information,” which is information on taste quality defined by us according to each sensor characteristic.

**Figure 13.**
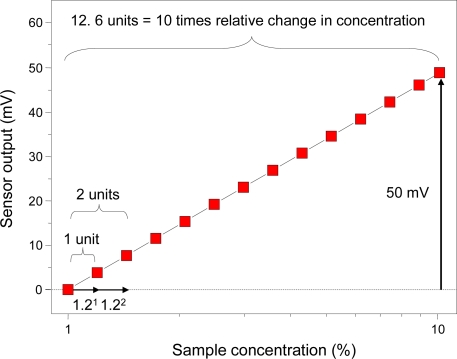
Example of conversion factor calculation for a taste sensor with slope of 50 mV/decade for some taste substance.

As a conversion example, imagine a taste sensor with a slope of 50 mV/decade for some taste substance (Figure 13). A 20% increment in the sample’s initial concentration of 1.0% is equal to a concentration of 1.2%.

This difference is the smallest that a person can distinguish and we define it as “1 unit”. Using this definition, a tenfold concentration difference is equal to 12.6 units, so the output is 3.96 mV/unit. The reciprocal or “conversion factor” is 0.25 unit/mV. Therefore, the taste information can be obtained by multiplying the conversion factor and the sensor output. For example, if the saltiness sensor is converted to taste information based on the result of KCl concentration dependence, it is described as “saltiness”. This definition of a unit meets the third requirement for a clearly defined unit of information from the taste sensor, allowing a clear understanding of the difference in taste intensity between samples.

Table 3 lists all the taste information provided by the Taste Sensing System (11 types of taste information from 8 taste sensors). All the information helps distinguish the difference in both taste quality and taste intensity between samples.

**Figure 14.**
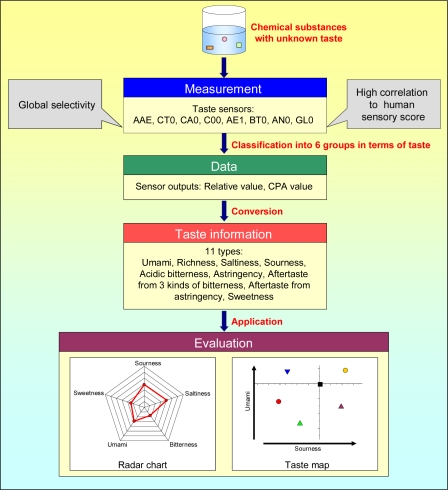
Flowchart from measurement to evaluation using taste sensors.

Figure 14 shows the flowchart from measurement to evaluation using taste sensors. First, chemical substances with an unknown taste are detected by taste sensors with global selectivity and high correlation to human sensory score. Second, sensor outputs, such as relative value and CPA value, are obtained and then converted to 11 types of taste information using pre-configured conversion factors. Last, the combination of taste information, radar chart, and taste map (see Subsection 5.1) are plotted to offer satisfactory results for taste qualities.

## 5. Applications

### 5.1. Taste evaluations for foods and beverages

**Figure 15.**
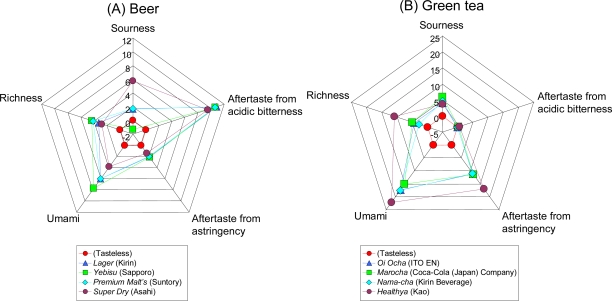
Radar charts for beer and green tea. In both radar charts, a reference solution of 30 mM KCl and 0.3 mM tartaric acid was used as a tasteless sample with taste information set to zero.

Taste sensors have applications in manufacturing of beverages, including beer [25,29,68,69], wine [79], green tea [52,80–82], sake [83,84], coffee [85,86], soybean paste [87], milk [88,89], and soy sauce [90], as well as in production of foodstuffs, such as rice [91], pork [92], and tomatoes [93]. The so-called “radar chart” is one method for understanding multivariate taste information at a glance. Figure 15 shows radar charts from taste sensors for beer and green tea, which are both sold mainly in Japan. The figures display taste information on “sourness,” “aftertaste from acidic bitterness”, “aftertaste from astringency,” “umami,” and “richness,” as described in Table 3.

All the taste information has the same meaning where the difference of 1 unit corresponds to the smallest taste difference that a person can distinguish. Also, the “reference solution” tasteless sample composed of 30 mM KCl and 0.3 mM tartaric acid was used with all related taste information set to zero. In this case, when the taste information value is 12.6, it is equivalent to the same degree of taste intensity as the concentration of the standard sample used for calculating the conversion factor in Table 3. For example, when the “saltiness” taste information is 12.6, the saltiness intensity is considered to be equivalent to 270 mM of potassium chloride. In other words, when a tasteless sample is used as the control, this analysis is an “absolute comparison.” Figure 15A shows that all beer samples have strong sourness, bitterness, and umami, clearly reflecting the taste of beer. In contrast, in Figure 15B, all green tea samples have strong astringency, umami, and richness, demonstrating that taste sensors provide explicit information on the taste of green tea.

**Figure 16.**
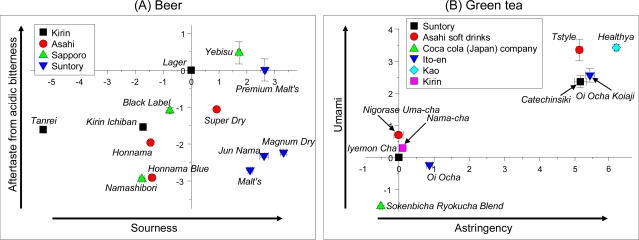
Taste maps for beer and green tea. Data are expressed as mean ±SD deviation (n = 4). In the two figures, *Lager* and *Iyemon Cha* were used as controls for beer and green tea, respectively. All taste information for each control sample was set to zero. All beers and green teas are on the Japanese market.

Another method for evaluating taste using taste sensors is a “taste map” using two kinds of taste information to evaluate taste. Figure 16 shows taste maps for beer and green tea. Samples, *Lager* and *Iyemon Cha*, were used as controls for beer and green tea, respectively, and all taste information for each control sample was set to zero. Therefore, when a product is used as control, this analysis is a “relative comparison”, unlike the “absolute comparison” described in Figure 15. All beer samples are easily distinguished from one another and the result is consistent with that of human taste scores. In addition, a taste map can show whether the taste difference between two samples is significant. For example, the *Honnama Blue* and *Namashibori* beers in Figure 16A measure nearly −1.5 for “sourness” and −3 for “aftertaste from acidic bitterness” but are within 1 unit for each of the information, meaning the bitterness and sourness of the two samples cannot be distinguished by people but the difference is significant for taste sensors. In contrast, green tea samples *Iyemon Cha* and *Healthya* are plotted more than 1 unit from each other on the two axes, meaning there is a significant difference in astringency and umami that any person can distinguish. The taste map is an extremely useful tool for comparing taste samples.

**Figure 17.**
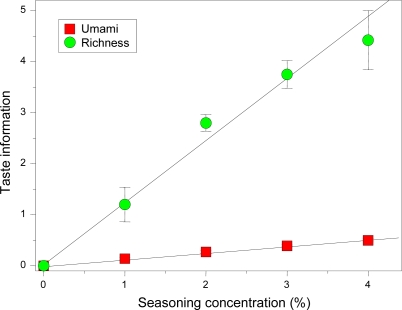
Effect of kokumi flavor on taste of commercial noodle soup base diluted threefold with pure water for measurement. A yeast extract (*Super Ye*, Ajinomoto, Co., Inc., Japan) was used as seasoning (Adapted from [95]).

The umami taste is an important quality because umami substances like amino acids and peptides are abundant in most foods, such as seaweed, bonito, oysters, beef, pork, tomatoes, soya beans, potatoes, and cheese. In addition, another flavor called “*kokumi*” produced by umami substances is the focus of recent attention; kokumi is a flavor that cannot be expressed as one of the five basic tastes, but is referred to as “continuity”, “mouthfulness” or “thickness”. One well-known kokumi substance is the tripeptide glutathione (γ-l-glutamyl-l-cysteinylglycine) [94]. We examined the kokumi flavor in noodle soup base using the taste information “umami” and “richness” (Figure 17).

A yeast extract (*Super Ye*, Ajinomoto, Co., Inc., Japan) containing glutathione was used as seasoning in noodle soup. This figure shows that “umami” was slightly increased by adding the seasoning, while “richness” increased dramatically. Although glutathione has no taste, it imparts kokumi to foods [94], and these taste sensors can evaluate this kokumi “flavor.” The taste information “richness” was calculated from the CPA value of the umami sensor AAE as described in Table 3. Little is known about how kokumi is perceived by living organisms but this result suggests it may be caused by adsorption of peptides on the tongue.

**Figure 18.**
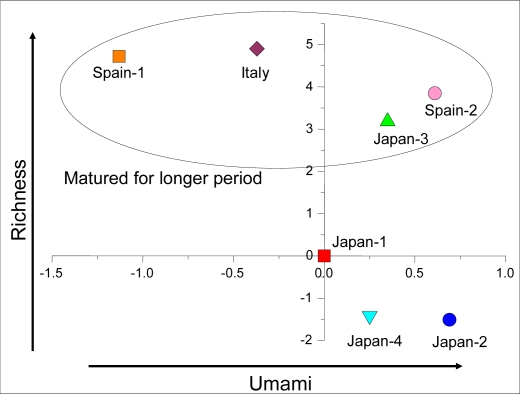
Taste map of Prosciutto ham. Four samples from Japan, two from Spain and one from Italy were measured. Each was mixed with the same amount of pure water and then stirred with a mixer for 1 minute. The solution was filtered through gauze and the filtrate measured as the sample.

Figure 18 shows the taste map using taste information “umami” and “richness” for Prosciutto ham. The four samples enclosed in the circle are matured for a longer period than other samples and had higher “richness” because more peptides are created by aging, which imparts more kokumi to these samples.

### 5.2. Quality control

**Figure 19.**
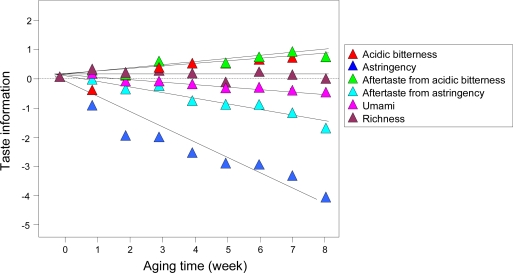
Change in taste qualities for green tea with aging. All green tea samples were stored in a temperature bath at 60 °C for up to 8 weeks (Adapted from [96]).

Food safety is the focus of consumer attention worldwide and the food and beverage industry requires strict quality control. Taste sensors can play an important role in the foodstuffs and beverages industry by detecting deteriorated taste qualities. Figure 19 shows changes in the taste of commercial PET-bottled green tea due to heat aging. Six types of taste information: “acidic bitterness”, “astringency”, “aftertaste from acidic bitterness”, “aftertaste from astringency”, “umami” and “richness” were measured, and all taste information for control samples without heat deterioration was set to zero. “Aftertaste from acidic bitterness” increased with aging, while “astringency” decreased. Astringency is usually an appreciated quality while bitterness is deprecated because it is not found in fresh tea. The results indicating deterioration of green tea with aging show how taste sensors can be effective in quality control.

**Figure 20.**
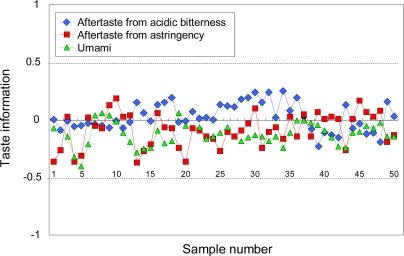
Differences between green tea lots (Adapted from [96]).

In addition to evaluating deterioration, taste sensors can detect differences between product lots. Figure 20 shows differences between 50 PET-bottled green tea lots based on taste information “bitterness,” “astringency,” and “umami”. All taste information for a green tea control sample of a given standard quality was set to zero. The results for all three types of taste information are all within 1 unit, indicating people would be unable to distinguish any differences between the 50 product lots.

### 5.3. Suppression effect

Taste substances interact with each other, increasing or decreasing the intensity of the six taste qualities, including astringency. This is called the synergistic/suppression effect. As a result, even when all taste materials in a product have been quantified by chemical analysis, the actual taste still cannot be evaluated, explaining why a taste sensor must detect interactions between taste substances. This section describes some examples. The foodstuffs industry uses many edible oils for various reasons, such as improving flavor, protecting from cooking heat, making more palatable, and increasing kinetic stability [97].

**Figure 21.**
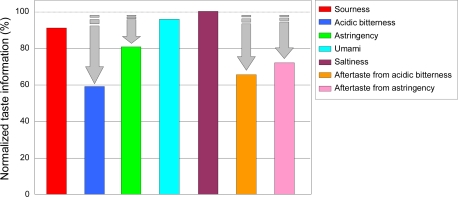
Suppression effect of taste qualities by edible oil (Adapted from [95]).

We examined the effect of one edible oil on several taste qualities using taste sensors (Figure 21). Salty, sour, umami, bitter, and astringent samples were made using 270 mM potassium chloride, 0.27 mM tartaric acid, 10 mM MSG, 0.01 vol% iso-alpha acid, and 0.05% tannic acid, respectively. Commercial *Nisshin Salad Oil* (Nisshin OilliO Group, Ltd., Japan) was added to each sample to give a final concentration of 0.1%, and homogenized (*homomixer f-model*, Tokusyu Kika Kogyo, Co., Ltd., Japan) at 10,000 rpm for 1 minute. Figure 21 shows addition of oil hardly changes “sourness”, “umami” and “saltiness” but greatly decreased “acidic bitterness”, “astringency”, “aftertaste from acidic bitterness” and “aftertaste from astringency.” This suggests that oil selectively suppresses bitterness and astringency, making foods taste milder.

In the pharmaceutical industry, evaluating the bitterness of drug products is very important because almost all active pharmaceutical ingredients in drug products are bitter. Therefore, drugs are usually formulated with sweeteners, such as sucrose, to suppress bitterness. Taste sensors can be used to evaluate both drug bitterness [70,98–107] and bitterness suppression effects [108–118]. Further, taste sensors are presently being studied for specificity, linearity, range, accuracy, precision, detection limit, quantitation limit and robustness for drug products [119], according to International Conference on Harmonisation (ICH) guideline Q2 [120].

**Figure 22.**
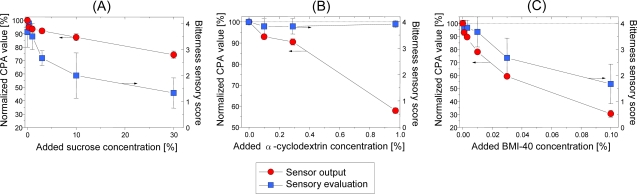
Bitterness suppression effect of bitter-masking materials on quinine hydrochloride using BT0 bitterness sensor. CPA values are normalized to 100, and expressed as mean ±SD (n = 4). The standard deviation for sensory evaluation score is the difference between volunteer taste panels (n = 3). All samples include 10 mM KCl as supporting electrolyte (Reprinted with permission from the Institute of Electrical Engineers of Japan [55]).

Figure 22 shows the bitterness suppression effect of the bitter-masking materials sucrose, α-cyclodextrin, and BMI-40 on quinine hydrochloride using the BT0 bitterness sensor [55]. Sucrose is a sweet substance used widely to suppress drug bitterness; α-cyclodextrin is a hydrophilic compound with a hydrophobic cavity and forms an inclusion complex by including hydrophobic compounds in the cavity [121]; BMI-40 (Kao Corporation, Japan) is composed mainly of phosphatidic acid and suppresses drug bitterness by “trap” and “masking” effects [122].

In Figure 22A, addition of sugars to quinine solution decreases CPA values by 20% as sucrose concentration increases, and sensory evaluation bitterness scores decrease greatly with addition of sugars. This suggests that the sensor can detect the suppression effect of sucrose. Addition of α-cyclodextrin greatly decreases the CPA value despite little decrease in the sensory evaluation score (Figure 22B). The sensory test has also confirmed that the bitterness sensory score of the quinine solution with addition of 9.7% α-cyclodextrin is decreased to 2.83, suggesting that α-cyclodextrin has low ability to suppress bitterness. This demonstrates that the sensor has a better ability to detect the suppression effect of α-cyclodextrin. With BMI-40, the CPA value decreases greatly with increasing BMI-40 concentration (Figure 22C), indicating that BMI-40 has the highest ability among the tested bitter-masking materials to suppress the bitterness of quinine hydrochloride. The corresponding decreased sensory evaluation score indicates a good agreement between the sensor and sensory evaluation score.

**Figure 23.**
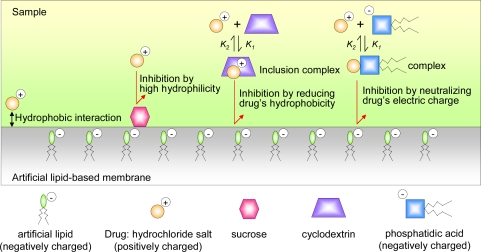
Possible mechanisms of suppression effect of bitter-masking materials (Reprinted with permission from the Institute of Electrical Engineers of Japan [55]).

Interestingly, this bitterness sensor does not respond to such bitter-masking materials. The sensor detects the suppression effect because it responds to drugs based on various interactions between the sensor and the bitter-masking materials. Figure 23 shows some possible mechanisms for sensor response to the suppression effect. This bitterness sensor has a negatively charged lipid that reacts strongly by hydrophobic interaction with the positively charged quinine hydrochloride. Sucrose does not interact directly with bitter substances, so it is believed to inhibit adsorption of bitter substances by the sensor by covering the sensor surface. As mentioned above, cyclodextrins interact selectively with bitter substances, so cyclodextrins are believed to inhibit the adsorption by inclusion. Since a CPA value cannot be observed for the BMI-40 solution (data not shown), which contains some phospholipids such as phosphatidylinositol, phosphatidic acid, phosphatidylethanolamine and phosphatidylcholine, it is considered to suppress bitterness by binding and neutralizing bitter substances in an aqueous solution, as shown in Figure 22. However, a negatively charged sensor immersed in a solution of BMI-60 (a similar product to BMI-40) also showed the suppression effect on the bitterness of quinine hydrochloride [93], suggesting that some of the phospholipids in the BMI-40 and BMI-60 suppresses bitterness by the partially covering on the sensor membrane.

## 6. Conclusions

In recent years, there has been more worldwide interest in the safety and quality of foods, beverages, and pharmaceuticals. Moreover, the global recession has increased price competition. To overcome the challenges, manufacturers must shorten the product cycle while offering higher quality at lower cost than competitors. Therefore, these market sectors require objective, rapid, accurate, and easy taste evaluation methods.

This review explains the principle of taste sensors based on artificial lipid and some commercial applications. Different from the earlier “electronic tongue,” our approach has been to develop taste sensors with global selectivity and high correlation with human sensory score. Sensors based on these unique features will play a key role in effective product development and quality control. Future developments are targeting development of a sweetness sensor, smaller equipment, and MEMS-based taste sensor chips [123,124].

## Figures and Tables

**Table 1.** Physicochemical properties of taste qualities.

**Taste quality**	**Adsorption ability**	**Taste threshold**
Saltiness	None	High
Sourness	None	Low
Umami	Low	Medium
Bitterness	High	Low

**Table 2.** Chemical components of taste sensors.

**Taste sensor**	**Artificial lipid**	**Plasticizer**
Umami sensor AAE	Phosphoric acid di(2-ethylhexyl) ester Trioctylmetylammonium chloride	Dioctyl phenylphosphonate
Saltiness sensor CT0	Tetradodecylammonium bromide 1-Hexadecanol	Dioctyl phenylphosphonate
Sourness sensor CA0	Phosphoric acid di(2-ethylhexyl) ester Oleic acid Trioctylmetylammomium chloride	Dioctyl phenylphosphonate
Bitterness sensor C00 (for acidic bitter materials)	Tetradodecylammonium bromide	2-Nitrophenyl octyl ether
Astringency sensor AE1	Tetradodecylammonium bromide	Dioctyl phenylphosphonate
Bitterness sensor BT0 (for bitter hydrochloride salts)	Phosphoric acid di-*n*-decyl ester	Bis(1-butylpentyl) adipate Tributyl *O*-acetylcitrate
Bitterness sensor AN0 (for basic bitter materials)	Phosphoric acid di-*n*-decyl ester	Dioctyl phenylphosphonate
Sweetness sensor GL0 (prototype)	Tetradodecylammonium bromide Gallic acid	Dioctyl phenylphosphonate

**Table 3.** Taste information converted from taste sensor outputs. The type of standard sample for calculating the conversion factor depends on the type of taste sensor.

**Taste sensor**	**Taste information from relative value**	**Taste information from CPA value**	**Standard sample for calculating conversion factor**
Umami sensor AAE	Umami	Richness	10 mM monosodium glutamate
Saltiness sensor CT0	Saltiness	(none)	270 mM potassium chloride
Sourness sensor CA0	Sourness	(none)	2.7 mM tartaric acid
Bitterness sensor C00	Acidic bitterness	Aftertaste from acidic bitterness	0. 01 vol% iso-alpha acid
Astringency sensor AE1	Astringency	Aftertaste from astringency	0.05% tannic acid
Bitterness sensor BT0	(none)	Aftertaste from hydrochloride salts	0.1 mM quinine hydrochloride
Bitterness sensor AN0	(none)	Aftertaste from basic bitterness	0.1 mM quinine hydrochloride
Sweetness sensor GL0	Sweetness	(none)	1 M sucrose

## References

[1] Legin A., Rudnitskaya A., Vlasov Y., Di Natale C., Davide F., D’Amico A. (1997). Tasting of beverages using an electronic tongue. Sens. Actuat. B.

[2] Vlasov Y., Legin A. (1998). Non-selective chemical sensors in analytical chemistry: from “electronic nose” to “electronic tongue”. Fresenius J. Anal. Chem.

[3] Legin A., Rudnitskaya A., Vlasov Y., Di Natale C., Mazzone E., D'Amico A. (1999). Application of electronic tongue for qualitative and quantitative analysis of mineral water and wine. Electroanal.

[4] Di Natale C., Paolesse R., Macagnano A., Mantini A., D'Amico A., Ubigli M., Legin A., Lvova L., Rudnitskaya A., Vlasov Y. (2000). Application of a combined artificial olfaction and taste system to the quantification of relevant compounds in red wine. Sens. Actuat. B.

[5] Vlasov Y., Legin A., Rudnitskaya A., Di Natale C., D’Amico A. (2005). Nonspecific sensor arrays (“electronic tongue”) for chemical analysis of liquids (IUPAC Technical Report). Pure Appl. Chem.

[6] Winquist F., Wide P., Lundström I. (1997). An electronic tongue based on voltammetry. Anal. Chim. Acta.

[7] Winquist F., Holmin S., Krantz-Rülcker C., Wide P., Lundström I. (2000). A hybrid electronic tongue. Anal. Chim. Acta.

[8] Toko K., Yamafuji K. (1980). Influence of monovalent and divalent cations on the surface area of phosphatidylglycerol monolayers. Chem. Phys. Lipids.

[9] Toko K., Nitta J., Yamafuji K. (1981). Dynamic aspect of a phase transition in DOPH-millipore membranes. J. Phys. Soc. Jpn.

[10] Toko K., Yamafuji K. (1981). Stabilization effect of protons and divalent cations on membrane structures of lipids. Biophys. Chem.

[11] Toko K., Ryu K., Ezaki S., Yamafuji K. (1982). Self-sustained oscillations of membrane potential in DOPH-millipore membranes. J. Phys. Soc. Jpn.

[12] Toko K., Tsukiji M., Ezaki S., Yamafuji K. (1984). Current-voltage characteristics and self-sustained oscillations in dioleyl phosphate-millipore membranes. Biophys. Chem.

[13] Toko K., Nosaka M., Tsukiji M., Yamafuji K. (1985). Dynamic property of membrane formation in a protoplasmic droplet of nitella. Biophys. Chem.

[14] Toko K., Yoshikawa K., Tsukiji M., Nosaka M., Yamafuji K. (1985). On the oscillatory phenomenon in an oil/water interface. Biophys. Chem.

[15] Toko K., Tsukiji M., Iiyama S., Yamafuji K. (1986). Self-sustained oscillations of electric potential in a model membrane. Biophys. Chem.

[16] Toko K., Nakashima N., Iiyama S., Yamafuji K. (1986). Self-oscillation of electric potential of a porous membrane impregnated with polymer multi-bilayer complexes. Chem. Lett.

[17] Iiyama S., Toko K., Yamafuji K. (1986). Effect of bitter substances on a model membrane system of taste reception. Agric. Biol. Chem.

[18] Iiyama S., Toko K., Yamafuji K. (1987). Electric oscillation in an excitable model membrane impregnated with lipid analogues. Biophys. Chem.

[19] Hayashi K., Yamafuji K., Toko K., Ozaki N., Yoshida T. (1989). Effect of taste substances on electric characteristics of a lipid cast membrane with a single pore. Sens. Actuat.

[20] Toko K., Hayashi K., Fujiyoshi T., Yamafuji K. (1989). Self-organized electric structure in uni- and multicellular biological systems. Synergetics.

[21] Iiyama S., Toko K., Hayashi K., Yamafuji K. (1989). Effect of several sweet substances on the electric characteristics of a dioleyl phosphate-millipore membrane. Agric. Biol. Chem.

[22] Hayashi K., Toko K., Yamafuji K. (1989). Effect of taste substances on aperiodic oscillation of an electric potential in a synthetic lipid membrane. Jpn. J. Appl. Phys.

[23] Hayashi K., Yamanaka T., Toko K., Yamafuji K. (1990). Multichannel taste sensor using lipid membranes. Sens. Actuat. B.

[24] Toko K., Yamanaka T., Hayashi K., Yamafuji K. (1990). Multi-channel taste sensor with lipid membranes.

[25] Ikezaki H., Hayashi K., Yamanaka M., Tatsukawa R., Toko K., Yamafuji K. (1991). Multichannel taste sensor with artificial lipid membrane. Trans. JEICE Jpn.

[26] Ikezaki H., Toko K., Hayashi K., Toukubo R., Yamanaka T., Sato K., Yamafuji K. Intelligent multi-channel taste sensor with lipid membranes.

[27] Ikezaki H., Toko K., Hayashi K., Toukubo R., Sato K., Yamafuji K. Taste sensing system with lipid membranes.

[28] Murata T., Hayashi K., Toko K., Ikezaki H., Sato K., Toukubo R., Yamafuji K. (1992). Quantification of sourness and saltiness using a multichannel sensor with lipid membranes. Sens. Mater.

[29] Toko K., Matsuno T., Yamafuji K., Hayashi K., Ikezaki H., Sato K., Toukubo R., Kawarai S. (1994). Multichannel taste sensor using electrical potential changes in lipid membranes. Biosens. Bioelectron.

[30] Hayashi K., Toko K., Yamanaka M., Yoshihara H., Yamafuji K., Ikezaki H., Toukubo R., Sato K. (1995). Electric characteristics of lipid-modified monolayer membranes for taste sensor. Sens. Actuat. B.

[31] Toko K. (2000). Biomimetic Sensor Technology.

[32] Bartoshuk L.M. (1975). Taste mixtures: is mixture suppression related to compression?. Physiol. Behav.

[33] Ninomiya Y., Funakoshi M., Kawamura Y., Kare M.R. (1987). Qualitative discrimination among umami and the four basic taste substances in mice. Umami: A Basic Taste.

[34] Kawamura Y., Funakoshi M., Kasahara Y., Yamamoto T. (1969). A neurophysiological study on astringent taste. Jpn. J. Physiol.

[35] Schiffman S.S., Suggs M.S., Sostman A.L., Simon S.A. (1992). Chorda tympani and lingual nerve responses to astringent compounds in rodents. Physiol. Behav.

[36] Bajec M.R., Pickering G.J. (2008). Astringency: mechanisms and perception. Crit. Rev. Food Sci. Nutr.

[37] Singer S.J., Nicolson G.L. (1972). The fluid mosaic model of the structure of cell membranes. Science.

[38] Chandrashekar J., Hoon M.A., Ryba N.J., Zuker C.S. (2006). The receptors and cells for mammalian taste. Nature.

[39] Reed D., Nanthakumar E., North M., Bell C., Bartoshuk L., Price R. (1999). Localization of a gene for bitter-taste perception to human chromosome 5p15. Am. J. Hum.Genet.

[40] Chandrashekar J., Mueller K.L., Hoon M.A., Adler E., Feng L., Guo W., Zuker C.S., Ryba N.J. (2000). T2Rs function as bitter taste receptors. Cell.

[41] Ishimaru Y., Inada H., Kubota M., Zhuang H., Tominaga M., Matsunami H. (2006). Transient receptor potential family members PKD1L3 and PKD2L1 form a candidate sour taste receptor. Proc. Natl. Acad. Sci. USA.

[42] Ishii S., Misaka T., Kishi M., Kaga T., Ishimaru Y., Abe K. (2009). Acetic acid activates PKD1L3–PKD2L1 channel—A candidate sour taste receptor. Biochem. Biophys. Res. Commun.

[43] Kellenberger S., Schild L. (2002). Epithelial sodium channel/degenerin family of ion channels: a variety of functions for a shared structure. Physiol. Rev.

[44] Lyall V., Heck G.L., Vinnikova A.K., Ghosh S., Phan T.H., Alam R.I., Russell O.F., Malik S.A., Bigbee J.W., DeSimone J.A. (2004). The mammalian amiloride-insensitive non-specific salt taste receptor is a vanilloid receptor-1 variant. J. Physiol.

[45] Nakashima K., Ninomiya Y. (1998). Increase in inositol 1,4,5-trisphosphate levels of the fungiform papilla in response to saccharin and bitter substances in mice. Cell Physiol. Biochem.

[46] Nakashima K., Ninomiya Y. (1999). Transduction for sweet taste of saccharin may involve both inositol 1,4,5-trisphosphate and cAMP pathways in the fungiform taste buds in C57BL mice. Cell Physiol. Biochem.

[47] DeSimone J.A., Lyalla V., Hecka G.L., Feldman G.M. (2001). Acid detection by taste receptor cells. Resp. Physiol.

[48] Yan W., Sunavala G., Rosenzweig S., Dasso M., Brand J.G., Spielman A.I. (2001). Bitter taste transduced by PLC-β_2_-dependent rise in IP_3_ and α-gustducin-dependent fall in cyclic nucleotides. Am. J. Physiol. Cell Physiol.

[49] Zhang Y., Hoon M.A., Chandrashekar J., Mueller K.L., Cook B., Wu D., Zuker C.S., Ryba N.J. (2003). Coding of sweet, bitter, and umami tastes: different receptor cells sharing similar signaling pathways. Cell.

[50] Kamo N., Miyake M., Kurihara K., Kobatake Y. (1974). Physicochemical studies of taste reception. I. Model membrane simulating taste receptor potential in response to stimuli of salts, acids and distilled water. Biochim. Biophys. Acta.

[51] Kamo N., Miyake M., Kurihara K., Kobatake Y. (1974). Physicochemical studies of taste reception. II. Possible mechanism of generation of taste receptor potential induced by salt stimuli. Biochim. Biophys. Acta.

[52] Ikezaki H., Taniguchi A., Toko K. (1997). Quantification of taste of green tea with taste sensor. Trans. IEE of Japan.

[53] Ikezaki H., Kobayashi Y., Toukubo R., Naito Y., Taniguchi A., Toko K. Techniques to control sensitivity and selectivity of multichannel taste sensor using lipid membranes.

[54] Ikezaki H., Naito Y., Kobayashi Y., Toukubo R., Taniguchi A., Toko K. (2000). Improvement of selectivity of taste sensor by control of charge density and hydrophobicity of lipid membrane. Technical Report of IEICE. OME.

[55] Kobayashi Y., Hamada H., Yamaguchi Y., Ikezaki H., Toko K. (2009). Development of an artificial lipid-based membrane sensor with high selectivity and sensitivity to the bitterness of drugs and with high correlation with sensory score. IEEJ Trans.

[56] Gouy M. (1910). Sur la constitution de la charge électrique à la surface d'un électrolyte. J. Phys. Theor. Appl.

[57] Chapman D.L. (1913). A contribution to the theory of electrocapillarity. Phil. Mag.

[58] Payens T.A.J. (1955). Ionized monolayers. Philips Res. Rep.

[59] Tyäuble H., Teubner M., Woolley P., Eibl H. (1976). Electrostatic interactions at charged lipid membranes. I. Effects of pH and univalent cations on membrane structure. Biophys. Chem.

[60] Oohira K., Toko K., Akiyama H., Yoshihara H., Yamafuji K. (1995). Electric characteristics of hybrid polymer membranes composed of two lipid species. J. Phys. Soc. Jpn.

[61] Oohira K., Toko K. (1996). Theory of electric characteristics of the lipid/PVC/DOPP membrane and PVC/DOPP membrane in response to taste stimuli. Biophys. Chem.

[62] Hayashi K., Shimoda H., Matsufuji S., Toko K. (1999). Adsorption of taste substances on lipid membranes of taste sensor. Trans. IEE of Japan.

[63] Iiyama S., Kuga H., Ezaki S., Hayashi K., Toko K. (2003). Peculiar change in membrane potential of taste sensor caused by umami substances. Sens. Actuat. B.

[64] Kumazawa T., Kashiwayanagi M., Kurihara K. (1985). Neuroblastoma cell as a model for a taste cell: mechanism of depolarization in response to various bitter substances. Brain Res.

[65] Danielsson L.G., Zhang Y.H. (1996). Methods for determining n-octanol-water partition constants. Trends Anal. Chem.

[66] Donovan S.F., Pescatore M.C. (2002). Method for measuring the logarithm of the octanol-water partition coefficient by using short octadecyl-poly (vinyl alcohol) high-performance liquid chromatography columns. J. Chromatogr. A.

[67] Gulyaeva N., Zaslavsky A., Lechner P., Chait. A., Zaslavsky B. (2003). pH dependence of the relative hydrophobicity and lipophilicity of amino acids and peptides measured by aqueous two-phase and octanol-buffer partitioning. J. Pept. Res.

[68] Gastl M., Hanke S., Back W. (2007). Analytical investigations to evaluate bitter sensation using a taste sensing system. Brew. Sci.

[69] Gastl M., Hanke S., Back W. (2008). “Drinkability”—balance and harmony of components as well as an incentive for continuing to drink. Brauwelt International.

[70] Okamoto M., Sunada H., Nakano M., Nishiyama R. (2009). Bitterness evaluation of orally disintegrating famotidine tablets using a taste sensor. Asian J. Pharm. Sci.

[71] Habara M., Chui H., Ikezaki H., Toko K. Detecting sweetness with lipid/polymer membranes.

[72] Habara M., Beppu D., Cui H., Ikezaki H., Toko K. (2007). Detecting of sugars using lipid/polymer membranes. Sens. Mater.

[73] Cui H., Habara M., Ikezaki H., Toko K. Study of surface-modified lipid/polymer membranes for detecting sweet taste substances.

[74] Stone H., Hayashi T. (1967). Gustatory responses to the L-amino acids in man. Olfaction and Taste II.

[75] Miura S., Sato S., Yoshida M., Kaneko T., Namba S., Kainosho M., Ichikawa K., Indow T., Sato S., Nonaka T., Noro K., Haga T., Yoshikawa S., Yoshida M. (1973). Taste and other sensations in mouth. Sensory Evaluation Handbook.

[76] Pfaffmann C., Field J. (1959). The sense of taste. Handbook of Physiology, Neurophysiology.

[77] Beider L.M., Beilder L.M. (1971). Part 2; Taste. Handbook of Sensory Physiology IV: Chemical Senses.

[78] Schutz H.G., Pilgrim F.J. (1957). Differential sensitivity in gustation. J. exp. Psychol.

[79] Baldacci S., Matsuno T., Toko K., Stella R., Rossi D.D. (1998). Discrimination of wine using taste and smell sensors. Sens. Mater.

[80] Chen R., Ikezaki H., Hayashi N., Kohata K., Kugimiya Y., Kobayashi K., Taniguchi A., Toko K. Study on evaluating jimi-taste of green tea using multichannel taste sensor.

[81] Hayashi N., Chen R., Ikezaki H., Yamaguchi S., Maruyama D., Yamaguchi Y., Ujihara T., Kohata K. (2006). Techniques for universal evaluation of astringency of green tea infusion by the use of a taste sensor system. Biosci. Biotechnol. Biochem.

[82] Hayashi N., Chen R., Ikezaki H., Ujihara T. (2008). Evaluation of the umami taste intensity of green tea by a taste sensor. J. Agric. Food Chem.

[83] Arikawa Y., Toko K., Ikezaki H., Shinha Y., Ito T., Oguri I., Baba S. (1995). Analysis of sake taste using multielectrode taste sensor. Sens. Mater.

[84] Arikawa Y., Toko K., Ikezaki H., Shinha Y., Ito T., Oguri I., Baba S. (1996). Analysis of sake mash using multichannel taste sensor. J. Ferment. Bioeng.

[85] Komai H., Naito Y., Sato K., Ikezaki H., Taniguchi A., Toko K. Measurement of coffee taste using lipid membrane taste sensors.

[86] Fukunaga T., Toko K., Mori S., Nakabayashi Y., Kanda M. (1996). Quantification of taste of coffee using sensor with global selectivity. Sens. Mater.

[87] Imamura T., Toko K., Yanagisawa S., Kume T. (1996). Monitoring of fermentation process of miso (soybean paste) using multichannel taste sensor. Sens. Actuat. B.

[88] Yamada H., Mizota Y., Toko K., Doi T. (1997). Highly sensitive discrimination of taste of milk with homogenization treatment using taste sensor. Mater. Sci. Eng.

[89] Mizota Y., Matsui H., Ikeda M., Ichihashi N., Iwatsuki K., Toko K. (2009). Flavor evaluation using taste sensor for UHT processed milk stored in cartons having different light permeabilities. Milchwissenschaft.

[90] Iiyama S., Yahiro M., Toko K. (2000). Measurements of soy sauce using taste sensor. Sens. Actuat. B.

[91] Thi U.T., Suzuki K., Okadome H., Homma S., Ohtsubo K. (2004). Analysis of the tastes of brown rice and milled rice with different milling yields using a taste sensing system. Food Chem.

[92] Sasaki K., Tani F., Sato K., Ikezaki H., Taniguchi A., Emori T., Iwaki F., Chikuni K., Mitsumoto M. (2005). Analysis of pork extracts by taste sensing system and the relationship between umami substances and sensor output. Sens. Mater.

[93] Chen R., Kobayashi Y., Ikezaki H., Taniguchi A., Toko K. Study of agricultural products using multichannel taste sensor with lipid/polymer membranes.

[94] Ueda Y., Yonemitsu M., Tsubuku T., Sakaguchi M., Miyajima R. (1997). Flavor characteristics of glutathione in raw and cooked foodstuffs. Biosci. Biotech. Biochem.

[95] Ikezaki H., Toko K., Uchida T. (2007). Taste Modification Technology of Food and Medicine.

[96] Ikezaki H. (2009). Monthly Food Plant Manager.

[97] Japan Oil Chemist’s Society (2001). The Handbook of Oil Chemistry-Lipids and Surfactants.

[98] Uchida T., Miyanaga Y., Tanaka H., Wada K., Kurosaki S., Ohki T., Yoshida M., Matsuyama K. (2000). Quantitative evaluation of the bitterness of commercial medicines using a taste sensor. Chem. Pharm. Bull.

[99] Uchida T., Kobayashi Y., Miyanaga Y., Toukubo R., Ikezaki H., Taniguchi A., Matsuyama K. (2001). A new method for evaluating the bitterness of medicines by semi-continuous measurement of adsorption using a taste sensor. Chem. Pharm. Bull.

[100] Miyanaga Y., Tanigake A., Nakamura T., Kobayashi Y., Ikezaki H., Taniguchi A., Matsuyama K., Uchida T. (2002). Prediction of the bitterness of single, binary- and multiple-component amino acid solutions using a taste sensor. Int. J. Pharm.

[101] Tanigake A., Miyanaga Y., Nakamura T., Tsuji E., Matsuyama K., Kunitomo M., Uchida T. (2003). The bitterness intensity of clarithromycin evaluated by a taste sensor. Chem. Pharm. Bull.

[102] Uchida T., Tanigake A., Miyanaga Y., Matsuyama K., Kunitomo M., Kobayashi Y., Ikezaki H., Taniguchi A. (2003). Evaluation of the bitterness of antibiotics using a taste sensor. J. Pharm. Pharmacol.

[103] Mukai J., Miyanaga Y., Ishizaka T., Asaka K., Nakai Y., Tsuji E., Uchida T. (2004). Quantitative taste evaluation of total enteral nutrients. Chem. Pharm. Bull.

[104] Ishizaka T., Miyanaga Y., Mukai J., Asaka K., Nakai Y., Tsuji E., Uchida T. (2004). Bitterness evaluation of medicines for pediatric use by a taste sensor. Chem. Pharm. Bull.

[105] Kataoka M., Miyanaga Y., Tsuji E., Uchida T. (2004). Evaluation of bottled nutritive drinks using a taste sensor. Int. J. Pharm.

[106] Tachiki H., Uchiyama H., Okuda Y., Uchida R., Kobayashi Y., Uchida T. (2005). Bitterness evaluation of famotidine orally disintegrating tablets using a taste sensor. Jpn. J. Med. Pharm. Sci.

[107] Kataoka M., Tokuyama E., Miyanaga Y., Uchida T. (2008). The taste sensory evaluation of medicinal plants and Chinese medicines. Int. J. Pharm.

[108] Takagi S., Toko K., Wada K., Yamada H., Toyoshima K. (2000). Detection of suppression of bitterness by sweet substance using a multichannel taste sensor. J. Pharma. Sci.

[109] Takagi S., Toko K., Wada K., Ohki T. (2001). Quantification of suppression of bitterness by phospholipids using taste sensor. J. Pharm. Sci.

[110] Nakamura T., Tanigake A., Miyanaga Y., Ogawa T., Akiyoshi T., Matsuyama K., Uchida T. (2002). The effect of various substances on the suppression of the bitterness of quinine–human gustatory sensation, binding, and taste sensor studies. Chem. Pharm. Bull.

[111] Miyanaga Y., Kobayashi Y., Ikezaki H., Taniguchi A., Uchida T. (2002). Bitterness prediction or bitterness suppression in human medicines using a taste sensor. Sens. Mater.

[112] Miyanaga Y., Inoue N., Ohnishi A., Fujisawa E., Yamaguchi M., Uchida T. (2003). Quantitative prediction of the bitterness suppression of elemental diets by various flavors using a taste sensor. Pharm. Res.

[113] Ogawa T., Nakamura T., Tsuji E., Miyanaga Y., Nakagawa H., Hirabayashi H., Uchida T. (2004). The combination effect of L-arginine and NaCl on bitterness suppression of amino acid solutions. Chem. Pharm. Bull.

[114] Miyanaga Y., Mukai J., Mukai T., Odomi M., Uchida T. (2004). Suppression of the bitterness of enteral nutrients using increased particle sizes of branched-chain amino acids (BCAAs) and various flavours: a taste sensor study. Chem. Pharm. Bull.

[115] Tsuji E., Uchida T., Fukui A., Fujii R., Sunada H. (2006). Evaluation of bitterness suppression of macrolide dry syrups by jellies. Chem. Pharm. Bull.

[116] Tokuyama E., Shibasaki T., Kawabe H., Mukai J., Okada S., Uchida T. (2006). Bitterness suppression of BCAA solutions by L-ornithine. Chem. Pharm. Bull.

[117] Hashimoto Y., Matsunaga C., Tokuyama E., Tsuji E., Uchida T., Okada H. (2007). The quantitative prediction of bitterness-suppressing effect of sweeteners on the bitterness of famotidine by sweetness-responsive sensor. Chem. Pharm. Bull.

[118] Ishizaka T., Okada S., Takemoto E., Tokuyama E., Tsuji E., Mukai J., Uchida T. (2007). The suppression of enhanced bitterness intensity of macrolide dry syrup mixed with an acidic powder. Chem. Pharm. Bull.

[119] Woertz K., Tissen C., Kleinebudde P., Breitkreutz J. (2010). Performance qualification of an electronic tongue based on ICH guideline Q2. J. Pharm. Biomat. Anal..

[120] ICH Expert Working Group Validation of analytical procedures: text and methodology Q2(R1). http://www.ich.org/LOB/media/MEDIA417.pdf.

[121] Uekama K. (2004). Design and evaluation of cyclodextrin-based drug formulation. Chem. Pharm. Bull.

[122] Katsuragi Y., Sugiura Y., Cao L., Otsuji K., Kurihara K. (1995). Selective inhibition of bitter taste of various drugs by lipoprotein. Pharmaceut. Res.

[123] Etoh S., Iwakura M., Nakashi K., Hattori R., Hayashi R., Toko K. Fabrication of taste sensor chip and portable taste sensor system.

[124] Etoh S., Feng L., Nakashi K., Hayashi K., Ishii A., Toko K. (2008). Taste sensor chip for portable taste sensor system. Sens. Mater.

